# Supplementary figures and images for: Reconstruction of the cell entry pathway of an extinct virus
Source: PLoS Pathog. 2018 Aug 6;14(8):e1007123. doi: 10.1371/journal.ppat.1007123 (PMC6095630; doi:10.1371/journal.ppat.1007123)

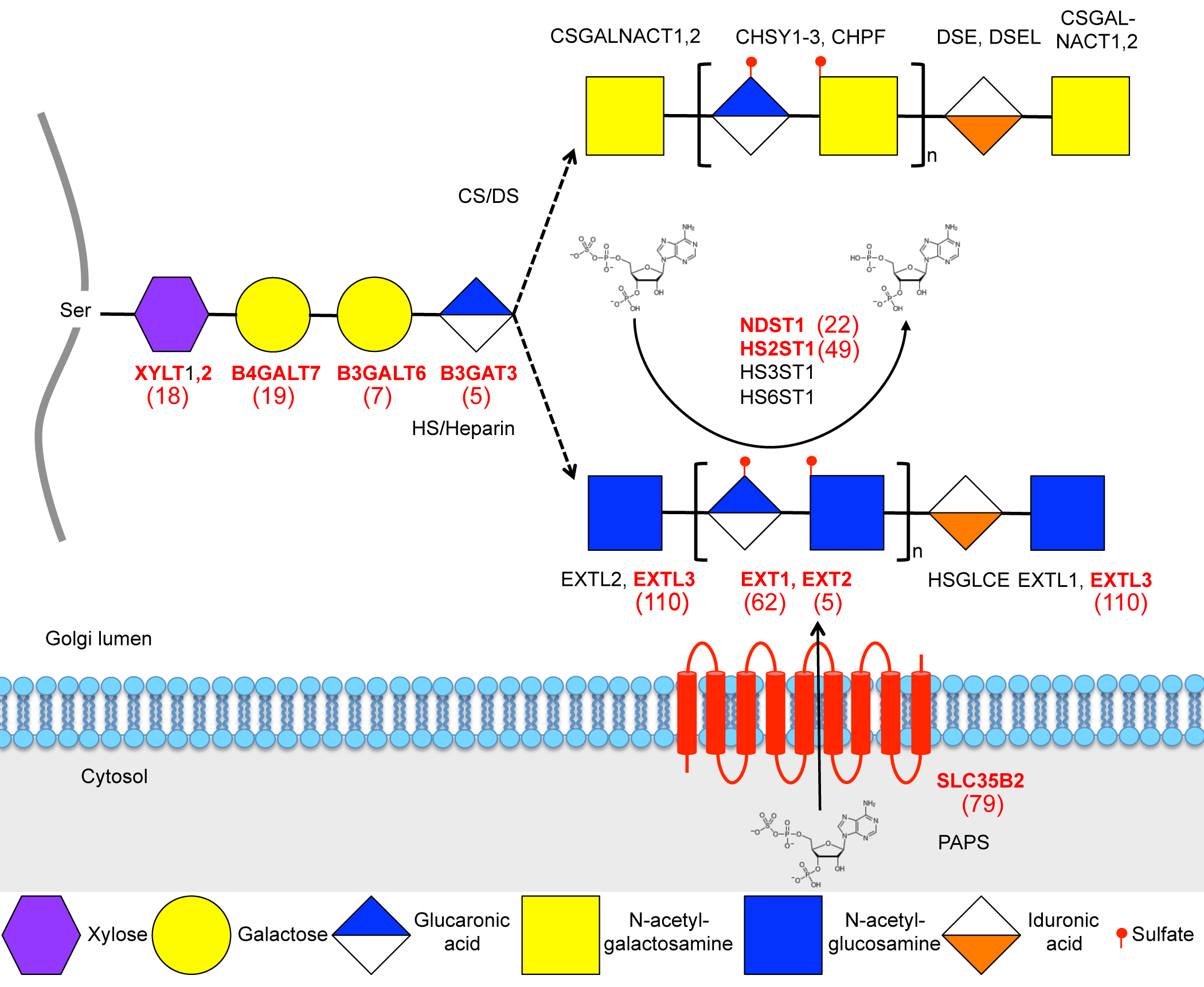

Supplement: S1 Fig — GAGs are added to a core protein (in grey). There is a core linkage of 4 sugars. The pathway then splits into the heparan sulfate/heparin pathway and the chondroitin sulfate/dermatan sulfate pathway. The enzymes that catalyze the sugar addition are written above/below the sugars. Sulfation is catalyzed by enzymes NDST1, HS2ST1, HS3ST1, and HS6ST1-3. Each enzyme adds a sulfate to a different position on the sugar. The sulfate donor, PAPS, is transported into the Golgi by SLC35B2. Genes highlighted in red were identified as hits in the haploid screen. The significance score for each hit, rounded to the nearest integer, is indicated in parentheses. (TIF) [file ppat.1007123.s001.tif]

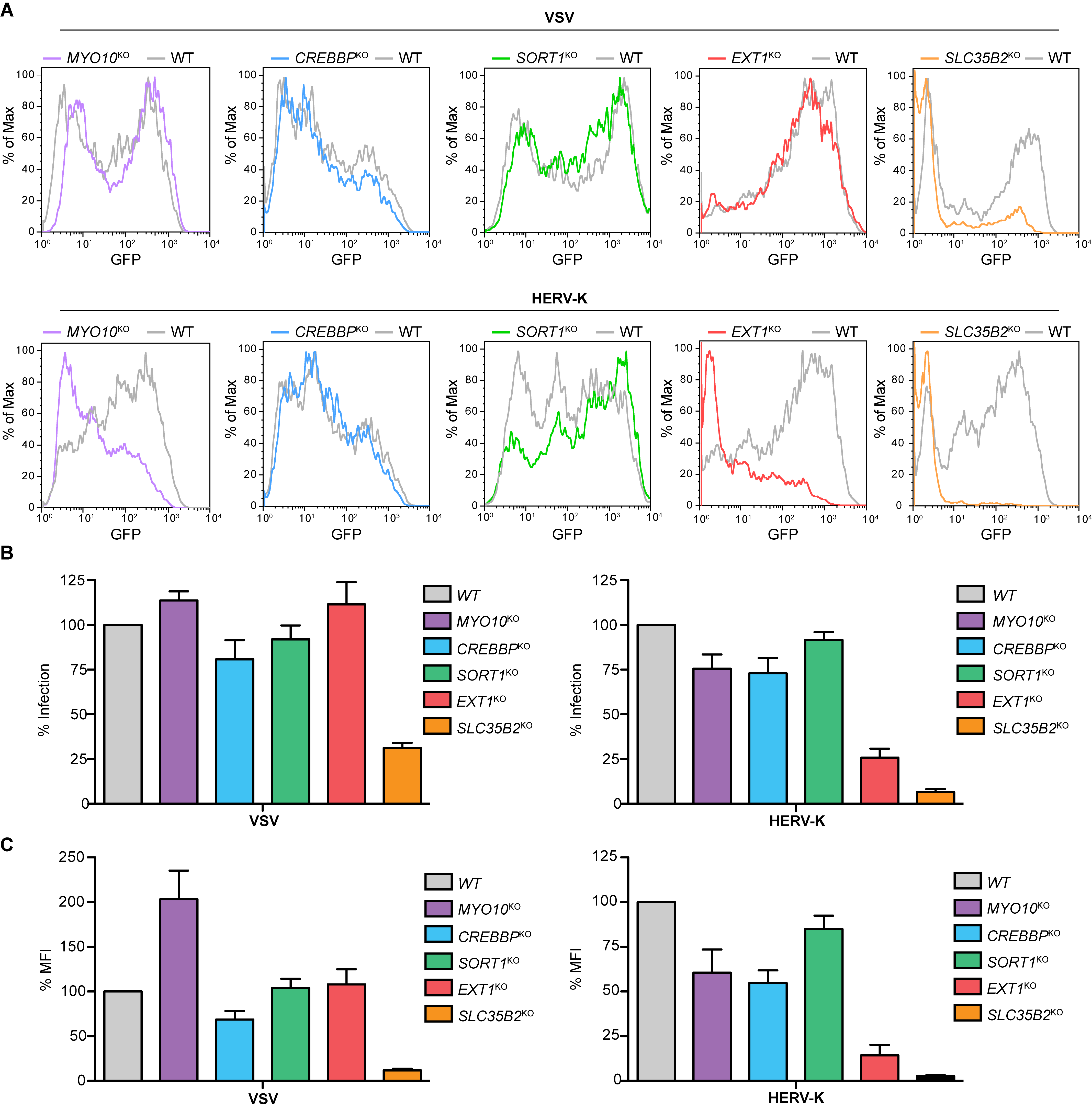

Supplement: S2 Fig — (A) Representative histograms from experiments in Fig 1D. Top: VSV infected cells. Bottom: VSV-HERVK infected cells. Histograms are from a single representative experiment. (B) Infectivity of VSV and VSV-HERVK in gene edited cells. Data are from the same experiment as Fig 1D and are normalized to infectivity in WT cells. (C) MFI of cells in (B). MFI of all cells for each condition are normalized to that of WT cells. (TIF) [file ppat.1007123.s002.tif]

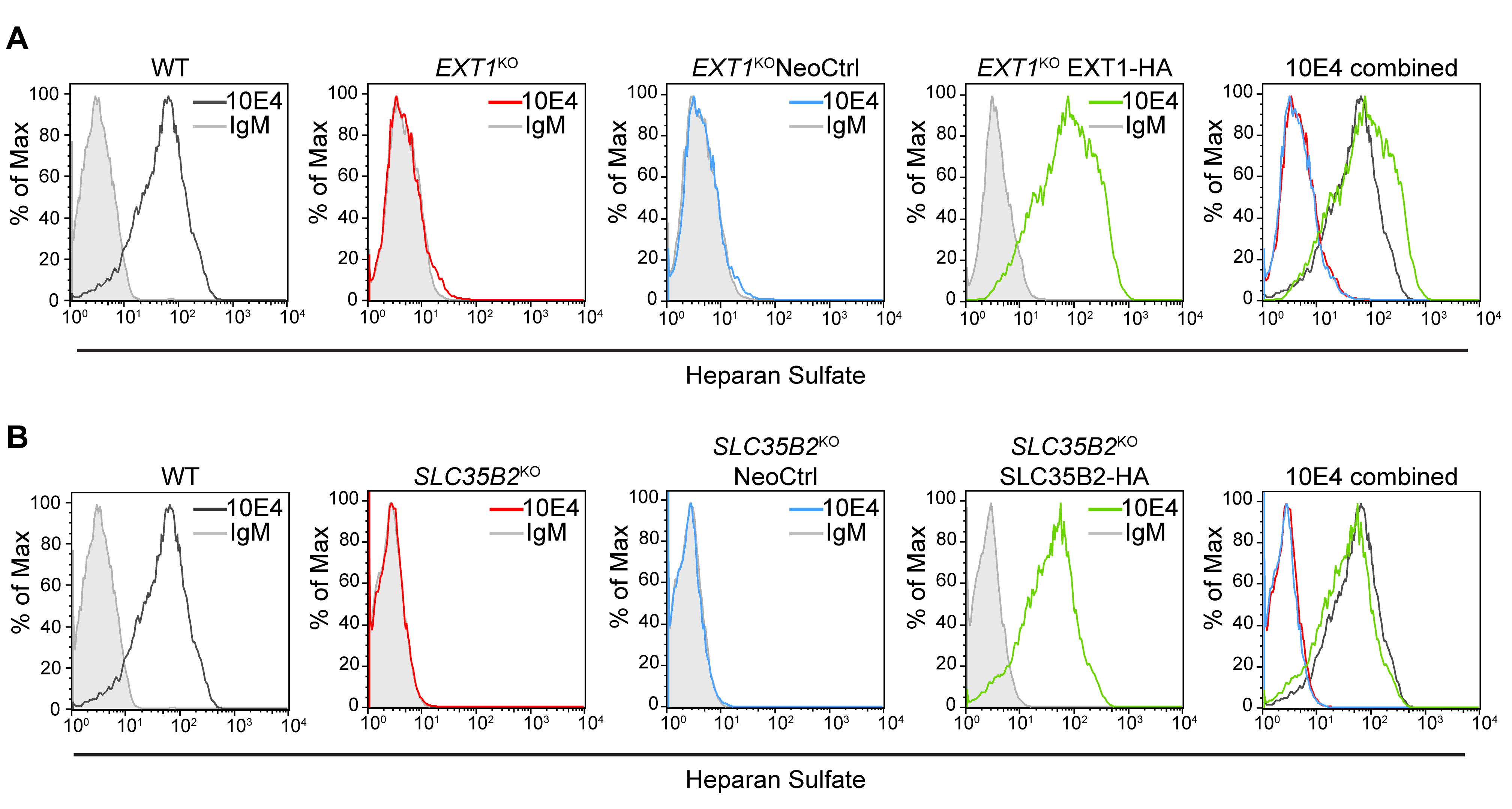

Supplement: S3 Fig — The indicated cell lines were stained with 10E4, a heparan sulfate-specific antibody, or mouse IgM isotype control antibody, and analyzed by flow cytometry. (A) WT HAP1, EXT1KO, EXT1KONeor Control, and EXT1KO+EXT1-HA cells. (B) WT HAP1, SLC35B2KO, SLC35B2KONeor Control, and SLC35B2KO+SLC35B2-HA cells. Representative histograms are shown from a single experiment. The WT histograms in (A) and (B) are the same sample and therefore identical. (TIF) [file ppat.1007123.s003.tif]

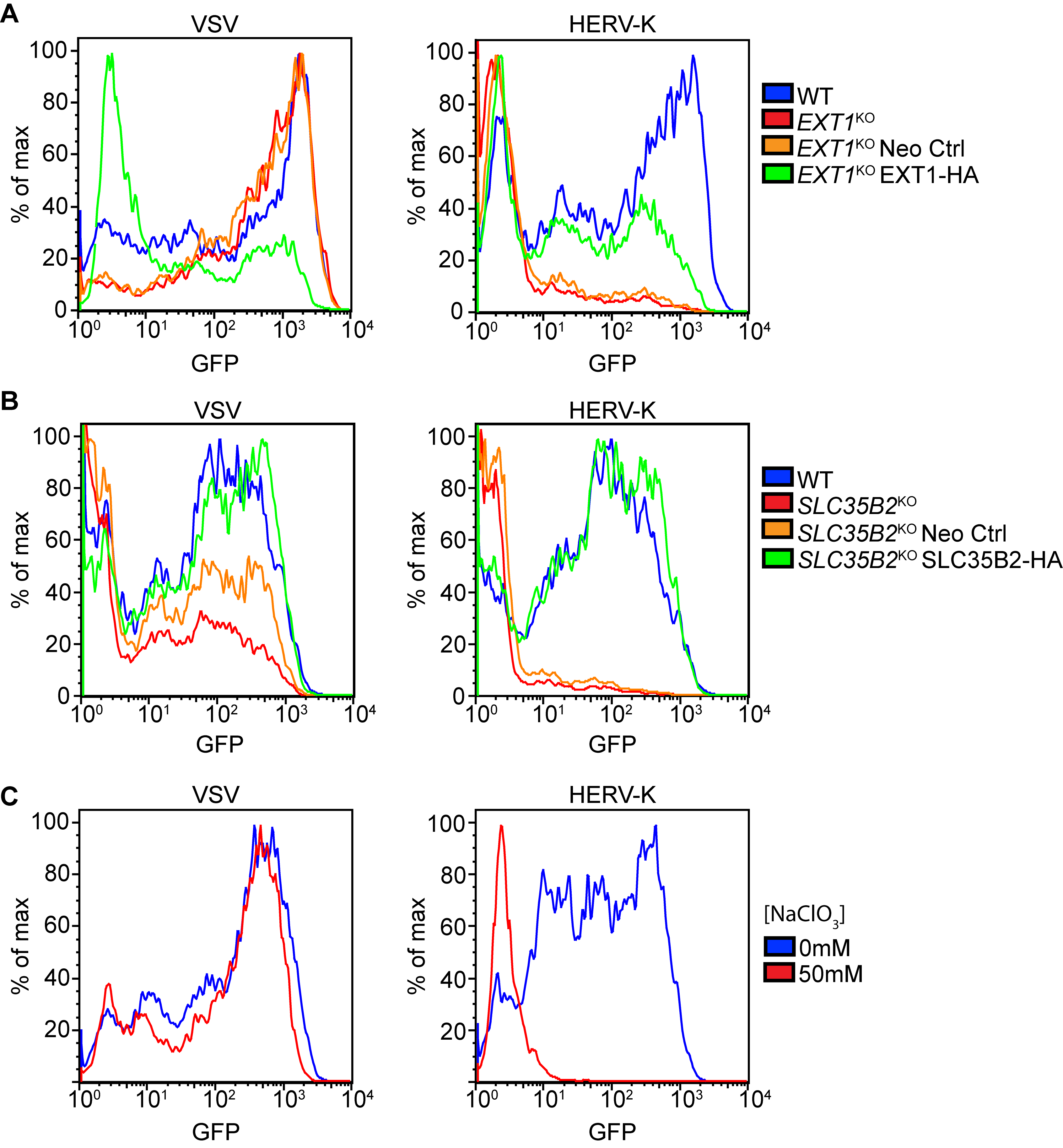

Supplement: S4 Fig — Representative histograms are shown from experiments in Fig 2A, 2B and 2C. (A) EXT1KO cells. (B) SLC35B2KO cells. (C) Sodium chlorate treated cells. Representative histograms are shown from a single experiment. (TIF) [file ppat.1007123.s004.tif]

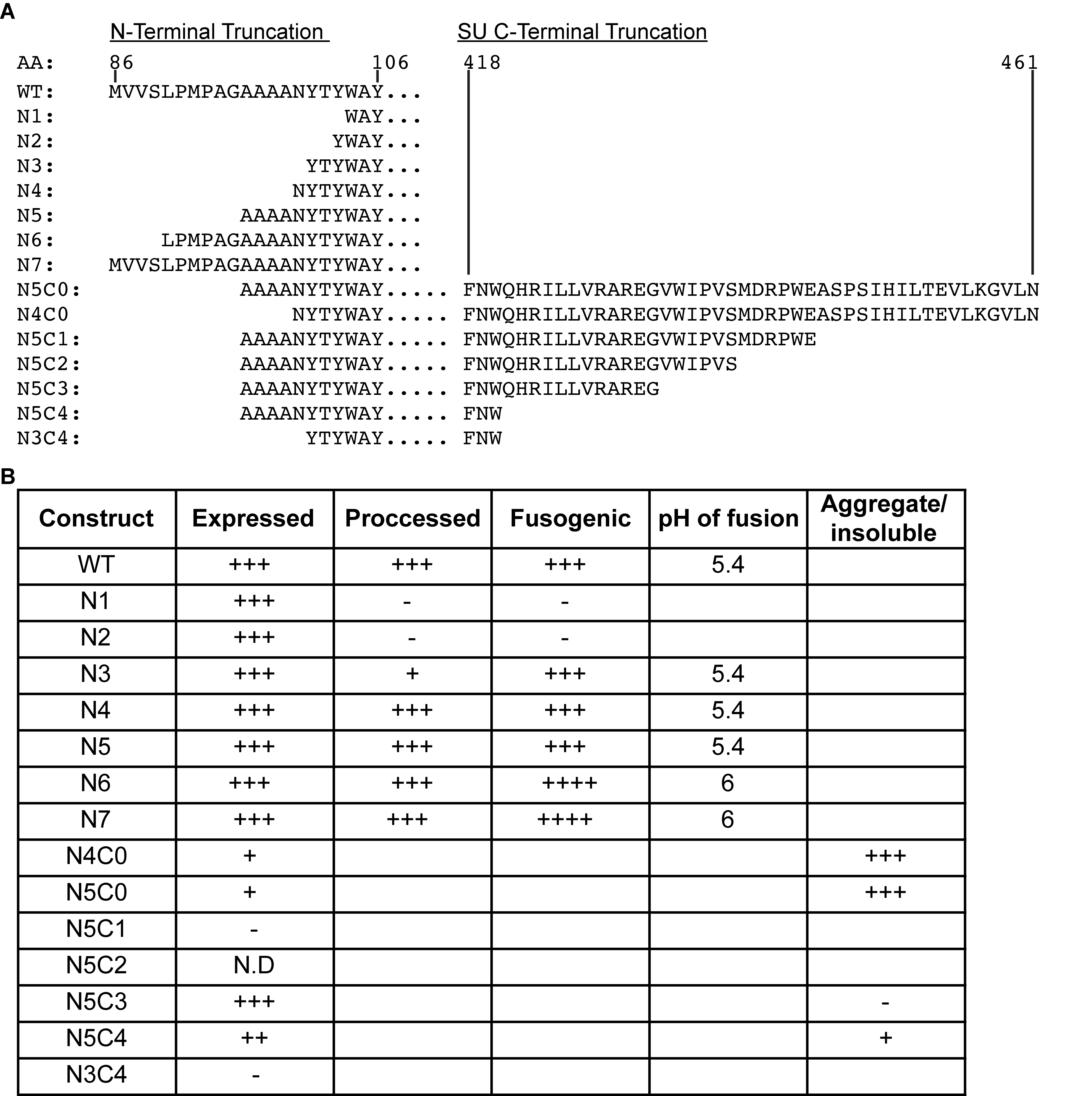

Supplement: S5 Fig — (A) Schematic of the sequences of the various N- and C-terminal truncations tested. The tissue plasminogen activator signal peptide was introduced at the N-terminus of all of the truncations. N1-N7 were made in otherwise full-length sequences. C-terminal truncations were further modified with an HA-tag, a 3C protease cleavage site, and a tandem His8X-His6X tag. Amino acid residue numbers are indicated above the sequences, with 1 being the initiating methionine. (B) Characteristics of HERV-K Env truncations. N1-N7 were expressed in BSRT7 cells and tested by Western blot for expression and proteolytic processing, and by cell-cell fusion assay for fusogenicity and pH dependence. C-terminal truncations were expressed in 293T cells. Protein in supernatant was isolated over cobalt resin and tested for expression, solubility and oligomerization state. N.D.: Not determined. Empty boxes: Assay not applicable to given construct. +: 1–30% of WT levels. ++: 31–60% of WT levels. +++ 61–100% of WT levels. ++++: 101–130% of WT levels. For C-terminal truncations, values are compared to N5C3. pH of fusion: Highest pH at which cell-cell fusion was observed. (TIF) [file ppat.1007123.s005.tif]

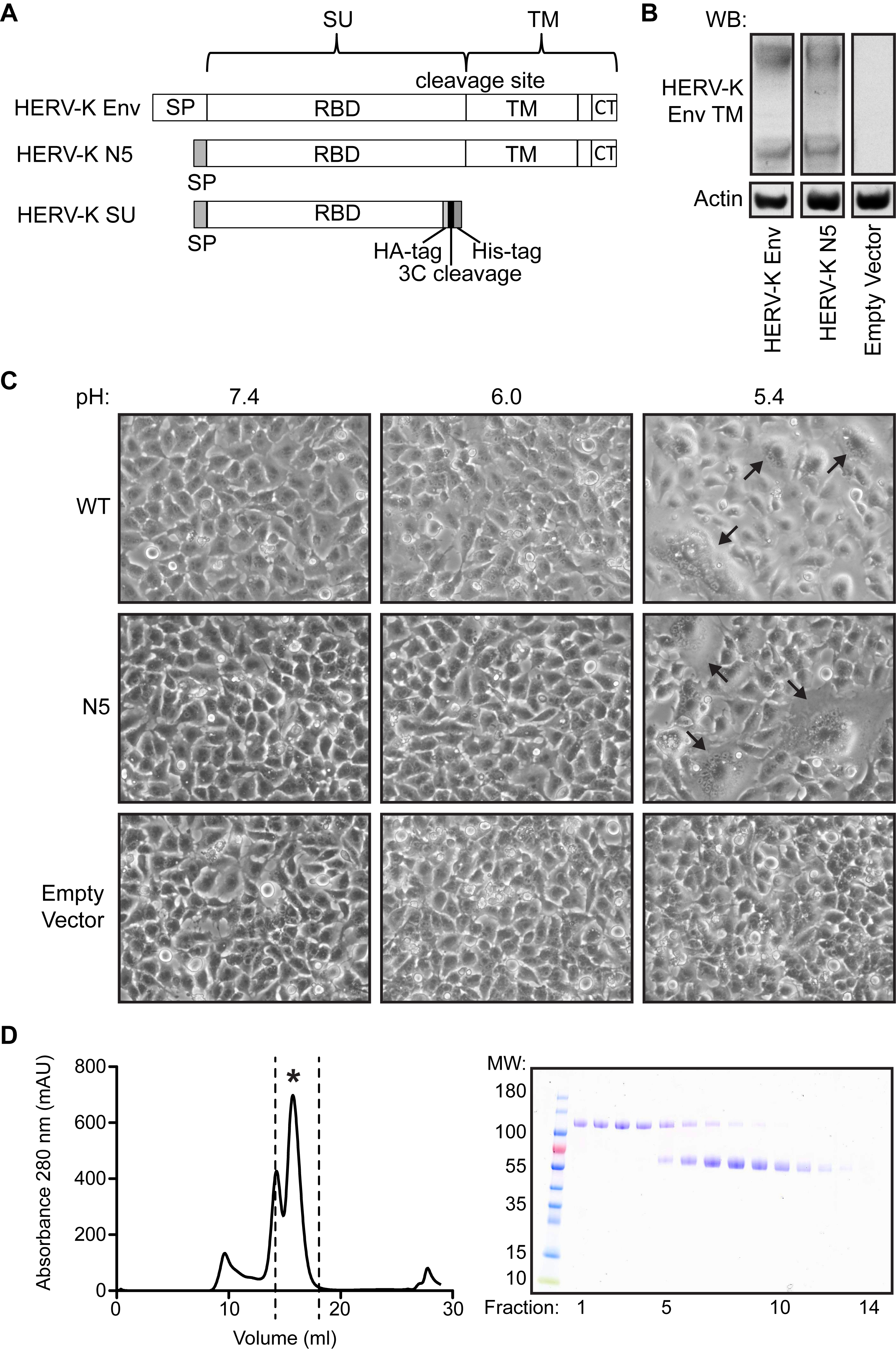

Supplement: S6 Fig — (A) Schematic of HERV-K Env, HERV-K N-terminal truncation N5, and HERV-K SU used in this study. (B) HERV-K Env and HERV-K N5 were transfected into BSRT7 cells and cell lysates were subjected to Western blot against HERV-K Env TM subunit and actin, to assess expression and proteolytic processing. For HERV-K Env blot: top band, uncleaved Env; bottom band, TM subunit. (C) BSRT7 cells were transfected with HERV-K Env, HERV-K N5, and empty vector. Cells were exposed to the indicated pH and assessed for the presence of multinucleated syncytia (indicated by arrows) (D) FPLC trace of HERV-K SU from gel filtration chromatography. The major peak (at approximately 15 ml, indicated with an asterisk) corresponds to monomeric SU. The peak at 13 ml corresponds to dimeric SU, and the peak at 9 ml is an aggregate of SU. Fractions from the FPLC (indicated with dashed lines) were run on a non-reducing SDS-PAGE and coomassie stained. Fraction 1 corresponds to 14.16 ml and fraction 14 corresponds to 18.06 ml. The top band at approximately 120 kDa represents the dimeric species and the lower band at approximately 60kDa represents monomer. Only fractions containing only monomer were used for pull-down experiments. (TIF) [file ppat.1007123.s006.tif]

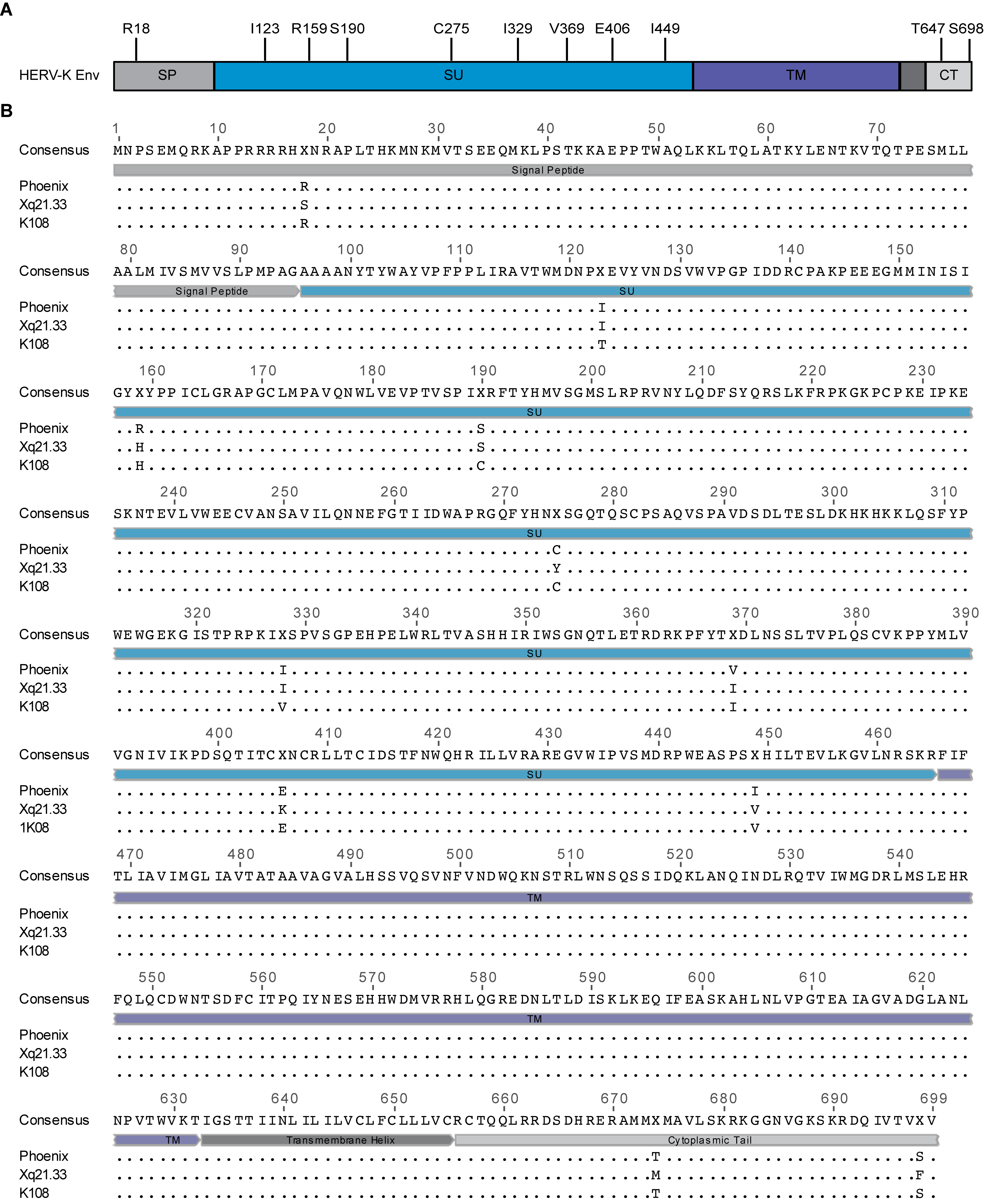

Supplement: S7 Fig — (A) Schematic of HERV-K Env. Positions of amino acids with differences between Phoenix and either Xq21.33 or K108 are shown with the amino acid identity in Phoenix indicated. SP: signal peptide. SU: surface subunit. TM: transmembrane subunit. CT: cytoplasmic tail. (B) Alignment of Phoenix, Xq21.33, and K108 Envs. (TIF) [file ppat.1007123.s007.tif]

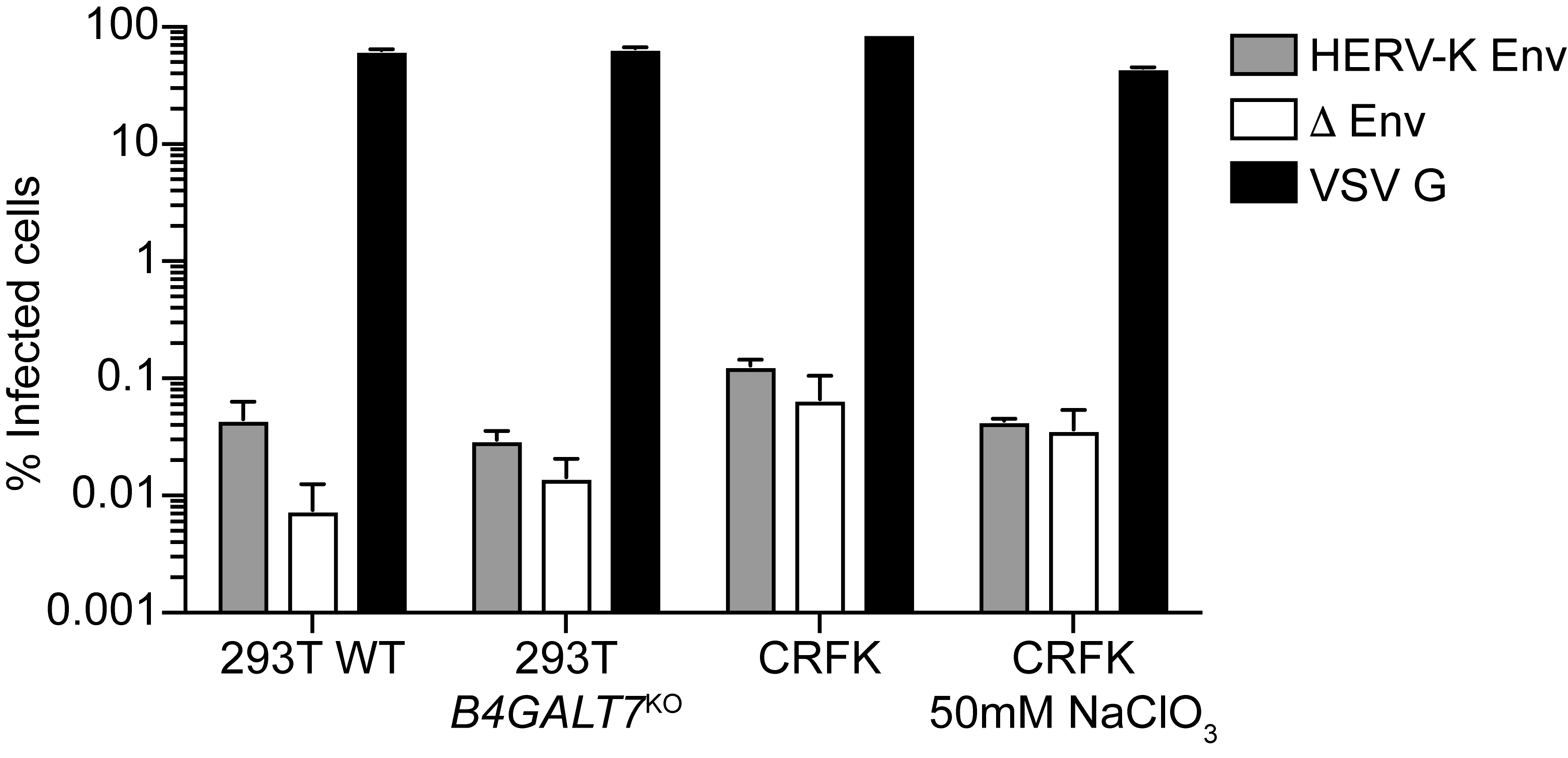

Supplement: S8 Fig — Lentivirus was produced as described above and particle concentration determined by p24 ELISA. The indicated cell lines were inoculated with equal particle numbers, based on p24 levels. % infected cells was determined by flow cytometry to determine the % GFP positive cells. Pseudotypes bearing HERV-K Env have an approximately 4-log defect in relative infectivity compared to those bearing VSV G, and have relative infectivities close to that of “bald” (Δ Env) particles. (TIF) [file ppat.1007123.s008.tif]
